# E3 ligase UHRF2 stabilizes the acetyltransferase TIP60 and regulates H3K9ac and H3K14ac via RING finger domain

**DOI:** 10.1007/s13238-016-0324-z

**Published:** 2016-10-14

**Authors:** Shengyuan Zeng, Yangyang Wang, Ting Zhang, Lu Bai, Yalan Wang, Changzhu Duan

**Affiliations:** 1 Department of Cell Biology and Medical Genetics, Molecular Medicine and Cancer Research Center, Chongqing Medical University, Chongqing, 400016 China; 2 Department of Obstetrics, The First Affiliated Hospital of Chongqing Medical University, Chongqing, 400016 China; 3 Key Laboratory of Medical Molecular Virology, School of Basic Medical Sciences, Shanghai Medical College, Fudan University, Shanghai, 200032 China; 4 Department of Pathology, Molecular Medicine and Cancer Research Center, Chongqing Medical University, Chongqing, 400016 China

**Keywords:** UHRF2, TIP60, ubiquitination, acetylation, hepatocellular carcinoma

## Abstract

UHRF2 is a ubiquitin-protein ligase E3 that regulates cell cycle, genomic stability and epigenetics. We conducted a co-immunoprecipitation assay and found that TIP60 and HDAC1 interact with UHRF2. We previously demonstrated that UHRF2 regulated H3K9ac and H3K14ac differentially in normal and cancer cells. However, the accurate signal transduction mechanisms were not clear. In this study, we found that TIP60 acted downstream of UHRF2 to regulate H3K9ac and H3K14ac expression. TIP60 is stabilized in normal cells by UHRF2 ubiquitination. However, TIP60 is destabilized in cancer cells. Depletion or inhibition of TIP60 disrupts the regulatory relationship between UHRF2, H3K9ac and H3K14ac. In summary, the findings suggest that UHRF2 mediated the post-translational modification of histones and the initiation and progression of cancer.

**Electronic supplementary material:**

The online version of this article (doi:10.1007/s13238-016-0324-z) contains supplementary material, which is available to authorized users.

## INTRODUCTION

Post-translational modifications (PTMs) of histones regulate gene expression. They are induced by several families of proteins including histone deacetylases (HDACs), histone acetyltransferases (HATs), physphorylases of the aurora kinases family, and histone ubiquitinases of the E3 ligase family (Basu et al., 2009; Pokholok et al., 2005; Tan et al., 2011; Wang et al., 2008). These modifications involving the interaction between histone and DNA regulate gene transcription either synergistically or antagonistically (Arnaudo and Garcia 2013; Su et al., 2016). UHRF2 is a multi-domain E3 ubiquitin ligase, which is related to mouse Np95 and human ICBP90. It comprises UBL, PHD, TTD, SET and RING-associated (SRA/YDG) and RING finger domains (Bronner et al., 2007; Qian et al., 2012; Wang et al., 2012). Several studies have demonstrated that UHRF2 represents a nodal point in the cell cycle network and regulates the epigenetics (Mori et al., 2011). UHRF2 interacts with the DNA methyltransferases (DNMT1, DNMT3a and DNMT3b), HDAC1, G9a and H3K9me2/me3 (Li et al., 2004; Mori et al., 2004; Pichler et al., 2011). Recently we found that UHRF2 influenced the acetylation of histone H3 on lysine 9 (H3K9ac) and lysine 14 (H3K14ac). However, the molecular mechanism was unclear. We investigated the mechanism underlying UHRF2-mediated regulation of H3K9ac and H3K14ac expression and the factors associated with differential regulation in normal and cancer cells.

Histone acetyltransferase TIP60 attracted our interest. It belongs to the MYST family and controls the acetylation of histone H2A on lysine 5 (H2AK5), histone H3 on lysine 14 (H3K14) and histone H4 (lysines 5, 8, 12, and 16) (Dai et al., 2013; Ikura et al., 2015; Jacquet et al., 2016; Renaud et al., 2016). TIP60 also participates in DNA repair, cellular growth, apoptosis and response to DNA double-strand breakage (Grezy et al., 2016; Jang et al., 2015; Sun et al., 2015). Recently, it was demonstrated that UHRF1 recruits histone acetyltransferase TIP60 and controls its expression and activity (Achour et al., 2009). In this study, the interactions between TIP60 and UHRF2 were detected using co-immunoprecipitation. It is suggested that TIP60 regulates H3K9ac and H3K14ac as downstream signal molecules.

We identified a previously undefined mechanism associated with TIP60-mediated UHRF2-regulation of H3K9ac and H3K14ac expression. In normal cells, UHRF2 overexpression enhances the expression of H3K9ac and H3K14ac, which is reversed in cancer cells. The regulatory mechanism is disrupted following deletion of the YDG or RING finger domains of UHRF2. In HEK293 cells, the interaction between UHRF2, TIP60 and HDAC1 was detected with co-immunoprecipitation, which was mediated via PHD and RING finger domains of UHRF2. Further, we found that TIP60 was a key intermediate in this molecular mechanism. Inhibition of TIP60 expression or activity disrupted the regulatory relationship. We also determined the levels of UHRF2, H3K9ac, H3K14ac, TIP60 and H2AK5ac in human hepatocellular carcinoma (HCC) tissues immunohistochemically. The results showed that all the aforementioned proteins were decreased under the high expression of UHRF2 in HCC tissues.

In summary, we identified a new mechanism underlying UHRF2-TIP60-H3K9ac and H3K14ac signaling axis. UHRF2 may contribute to the initiation and development of primary hepatocellular cancer by TIP60, which warrants further investigation.

## RESULTS

### UHRF2 regulates the expression of H3K9ac and H3K14ac

**Figure 1:**
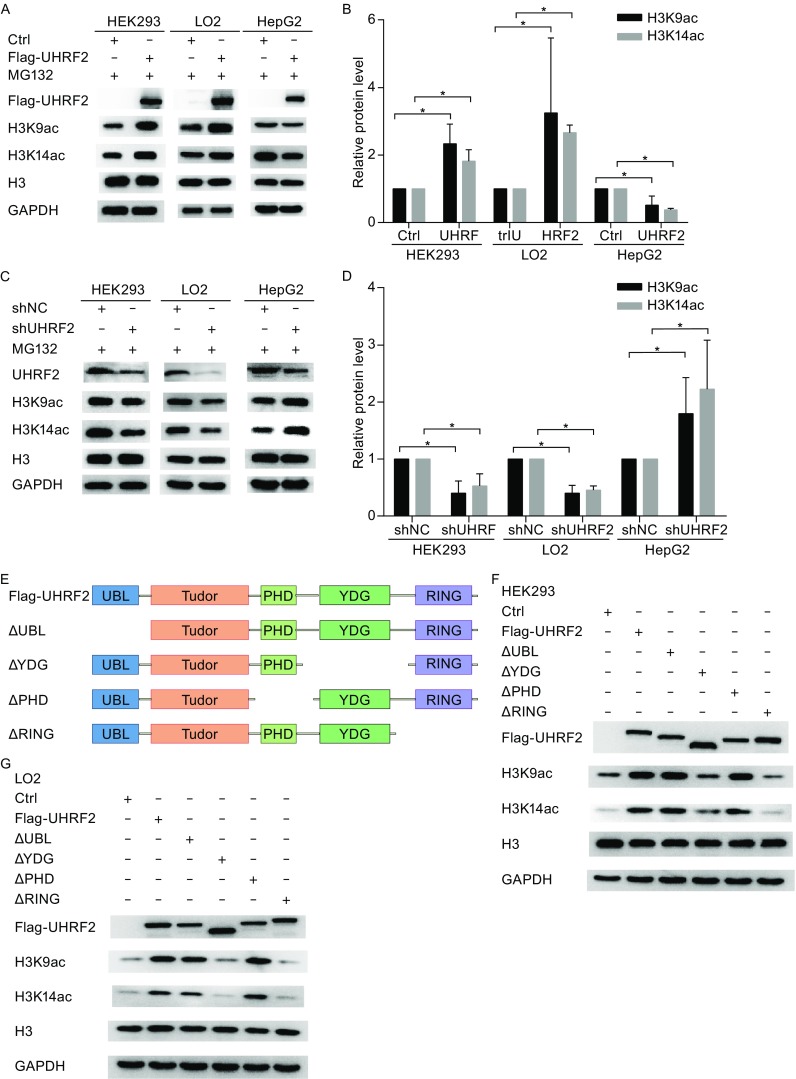

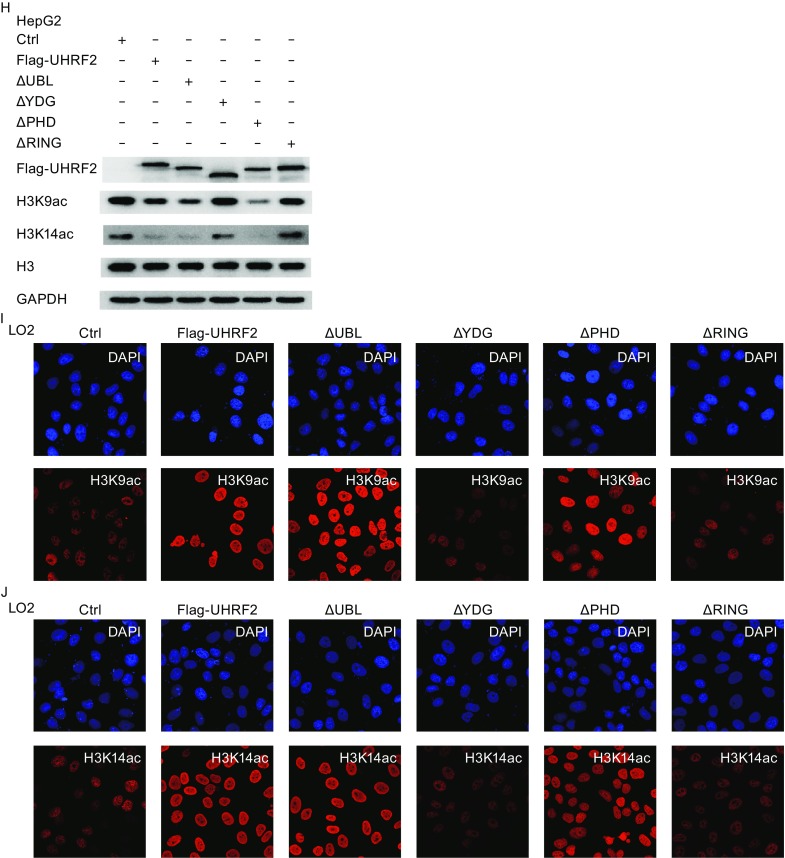
**UHRF2 regulates the expression of H3K9ac and H3K14ac**. (A) HEK293, LO2 and HepG2 cells were transfected with the indicated plasmids. Cellular lysates were analyzed by Western blot. UHRF2 was detected using anti-Flag antibodies. H3 and GAPDH were used as loading controls. (B) H3K9ac and H3K14ac levels were normalized to 1 at control groups. Significance was represented as *P < 0.05. (C and D) Endogenous UHRF2 protein levels were decreased by shRNA against UHRF2. The cellular lysates were analyzed by Western blot and quantified using Image J software. (E) Various UHRF2 deletion mutants; (F–H) HEK293, LO2 and HepG2 cells were transiently transfected with indicated plasmids. After 48 h, the cellular lysates were analyzed by Western blot. (I and J) Immunofluorescence analyses were performed using anti-H3K9ac or anti-H3K14ac antibodies

To determine the relationship between UHRF2, H3K9ac and H3K14ac, Western blot was performed. In normal cells (HEK293 and LO2 cells), UHRF2 overexpression elevated the expression of H3K9ac and H3K14ac compared with that of the control group. Opposite findings were observed in cancer cells (HepG2 cells) (Fig. 1A and 1B). Depletion of UHRF2 in HEK293 and LO2 cells lowered the expression of H3K9ac and H3K14ac compared with those of the control group. Similarly, HepG2 cells showed an inverse relationship (Fig. 1C and 1D) suggesting totally opposite results of the regulatory mechanism of UHRF2, H3K9ac and H3K14ac in different cells. Furthermore, we found that the YDG and RING finger domains of UHRF2 were required for this regulatory mechanism, based on Western blot and immunofluorescent assays (Fig. 1F–J). UHRF2 is an E3 ligase belonging to the RING-finger family, and the RING finger domain is required for E3 ligase activity. These results suggested that UHRF2 ligase activity was required for the regulatory mechanism.

### UHRF2 interacts with TIP60 and HDAC1

**Figure 2:**
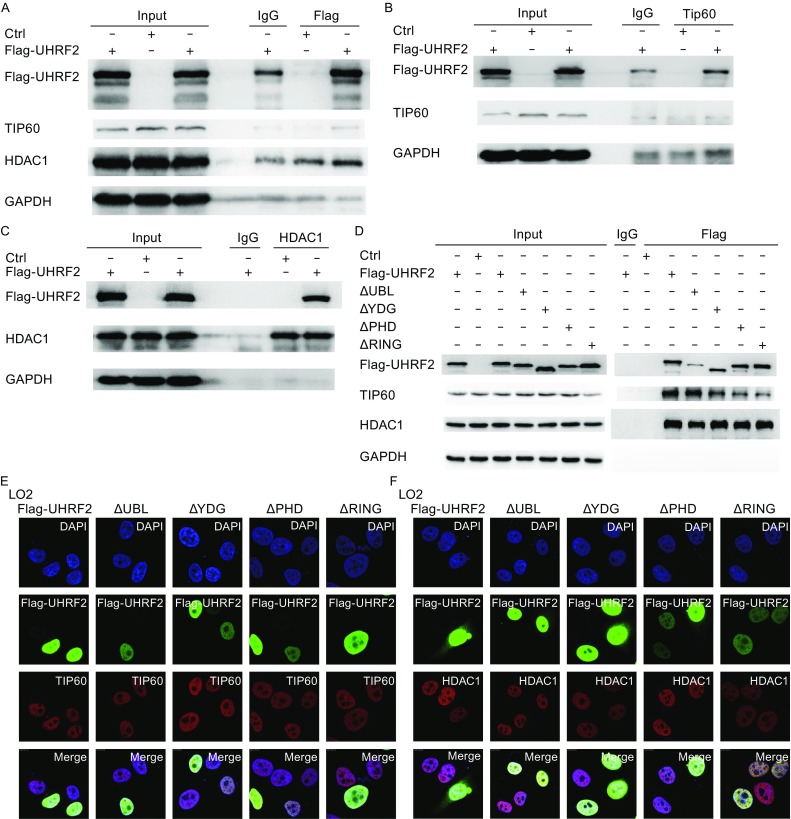
**UHRF2 interacts with TIP60 and HDAC1**. (A) HEK293 cells were transfected with control or Flag-UHRF2 plasmids, respectively. After 48 h, the total cellular lysates were immunoprecipitated using anti-Flag antibodies. (B) HEK293 cells were transfected with plasmids as shown. Cellular lysates were immunoprecipitated with anti-TIP60 antibodies and analyzed by Western blot. (C) HEK293 cells were transfected with the indicated plasmids. Total cellular lysates were subjected to HDAC1 immunoprecipitation. (D) HEK293 cells were transfected with full-length UHRF2 or various deletion mutant constructs of UHRF2 plasmids. Cellular lysates were immunoprecipitated using anti-Flag antibodies and analyzed by Western blot. (E and F) Double-labeling immunofluorescence combined with CLSM observation revealed co-localization of TIP60 and UHRF2 in LO2 cells. Co-localization of HDAC1 and UHRF2 was also revealed along with nuclear expression of all proteins

Recent studies confirmed that UHRF1 recruits TIP60 and controls its expression and activity (Achour et al., 2009). UHRF2 was implicated in epigenetics via association with DNMTs, G9a, HDAC1, H3K9me2/3 and hemi-methylated DNA (Mori et al., 2011; Pichler et al., 2011). Both UHRF2 and UHRF1 belong to the UHRF family and show 67% sequence similarity (Bronner et al., 2007). Hence, we hypothesized that UHRF2 regulates H3K9ac and H3K14ac expression probably via TIP60 or HDAC1. A co-immunoprecipitation assay was performed to establish the interaction between UHRF2, TIP60 and HDAC1. A significant TIP60 and HDAC1 immunoprecipitation was observed using Flag-UHRF2 (Fig. 2A). In Fig. 2B and 2C, the reciprocal co-immunoprecipitation was performed and the interaction between UHRF2, TIP60 and HDAC1 was verified. In Fig. 2D, we demonstrated that the PHD and RING finger domains of UHRF2 were key domains in the interaction. Deletion of the PHD or RING finger domain of UHRF2 disrupted the interaction between UHRF2 and TIP60. However, the interaction between UHRF2 and HDAC1 was not affected. The transformation was not caused by the mis-localization of the deletion mutants of UHRF2. Double immunofluorescent staining revealed co-localization of UHRF2, TIP60 and HDAC1 proteins in LO2 cells (Fig. 2E and 2F) suggesting that UHRF2 interacted with TIP60 and HDAC1, and the interaction between UHRF2 and TIP60 was induced via the PHD and RING finger domains of UHRF2.

### UHRF2 enhances the expression and activity of TIP60

**Figure 3:**
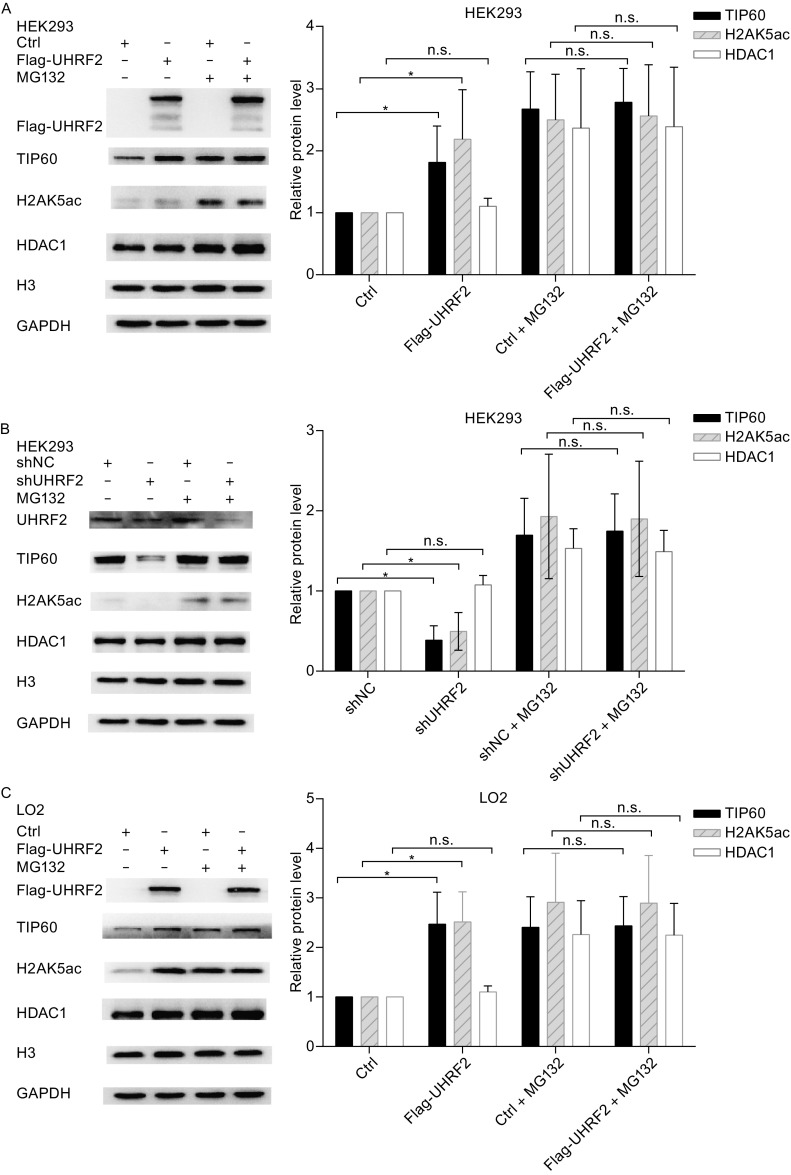

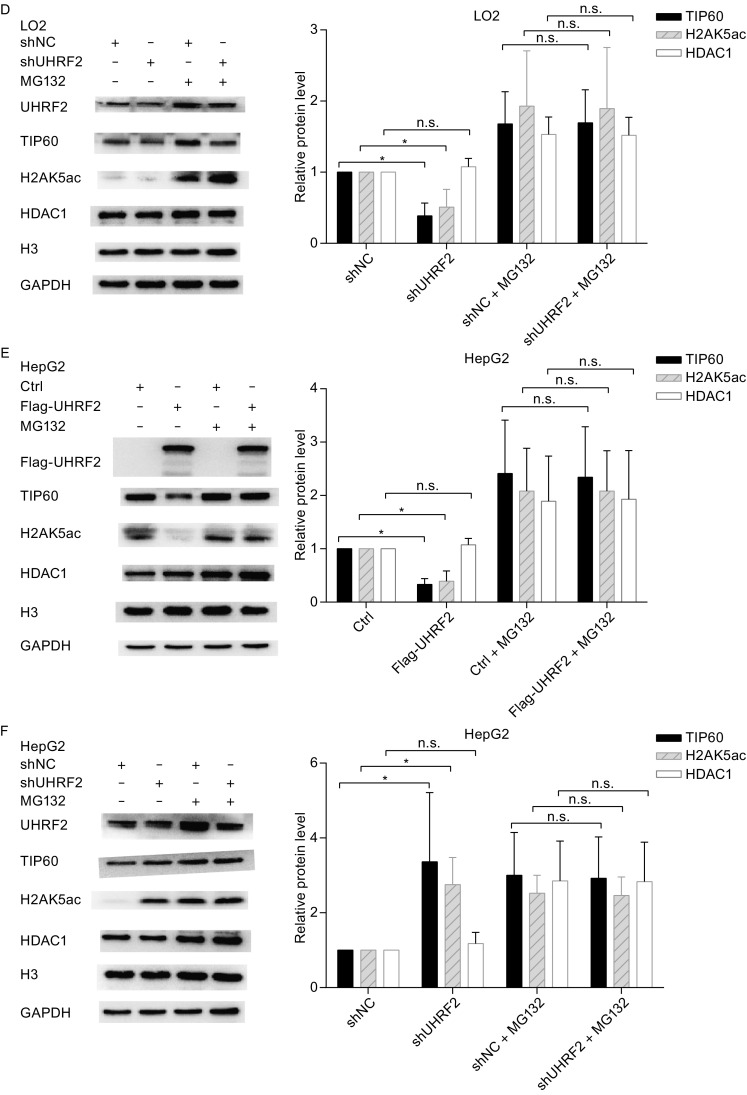

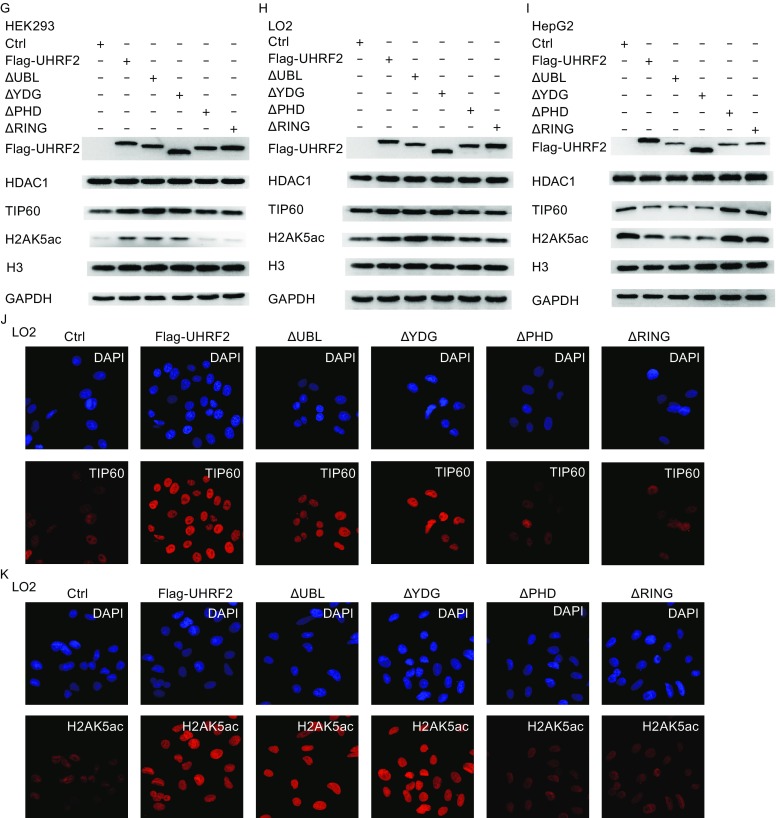
**UHRF2 regulates the expression and activity of TIP60**. (A, C and E) HEK293, LO2 and HepG2 cells were transfected with control or Flag-UHRF2 plasmids. They were exposed to MG132 (10 μg/mL) for 12 h and harvested. The cellular lysates were analyzed by Western blot. (B, D and F) HEK293, LO2 and HepG2 cells were transfected with shNC or shUHRF2. After 48 h, the total cellular lysates were analyzed by Western blot. Data were expressed as means ± SD, *n* = 3. Significance was indicated as *P < 0.05, and non-significance as n.s *P* > 0.05. (G–I) HEK293, LO2 and HepG2 cells were transfected with the plasmids as shown. The cellular lysates were analyzed by Western blot. (J and K) Immunofluorescence analyses were performed using anti-TIP60 or anti-HDAC1 antibodies

To address the physiological relevance of these interactions, we tested whether modulation of the cellular levels of UHRF2 affected the TIP60 levels and activity. Recent studies emphasized the role of TIP60 as a unique histone acetyltransferase for the acetylation of H2AK5, which is considered as an endogenous marker of TIP60 activity (Bhaumik et al., 2007). In Fig. 3A, 3C and 3E, UHRF2 overexpression in normal cells induced a significant increase of TIP60 levels and activity, which was reversed in cancer cells. However, no obvious alteration in HDAC1 protein levels was observed. These results suggested that the expression and activity of TIP60 were increased by UHRF2 in normal cells, but decreased in cancer cells. In addition, we employed shRNA to suppress endogenous UHRF2. The results showed that ablation of endogenous UHRF2 decreased TIP60 activity in normal cells, but increased in HepG2 cells. HDAC1 protein levels also showed no significant variation (Fig. 3B, 3D and 3F). Additional investigations showed that deletion of the PHD or RING finger domains of UHRF2 abolished the regulatory relationship (Fig. 3G-I), which suggested that PHD and RING finger domains of UHRF2 were the key domains in this mechanism. Furthermore, immunofluorescence staining showed similar results indicating that the PHD and RING finger domains of UHRF2 were pivotal domains (Fig. 3J and 3K) and that the ligase activity of UHRF2 was required.

### UHRF2 ubiquitinates and stabilizes TIP60

**Figure 4:**
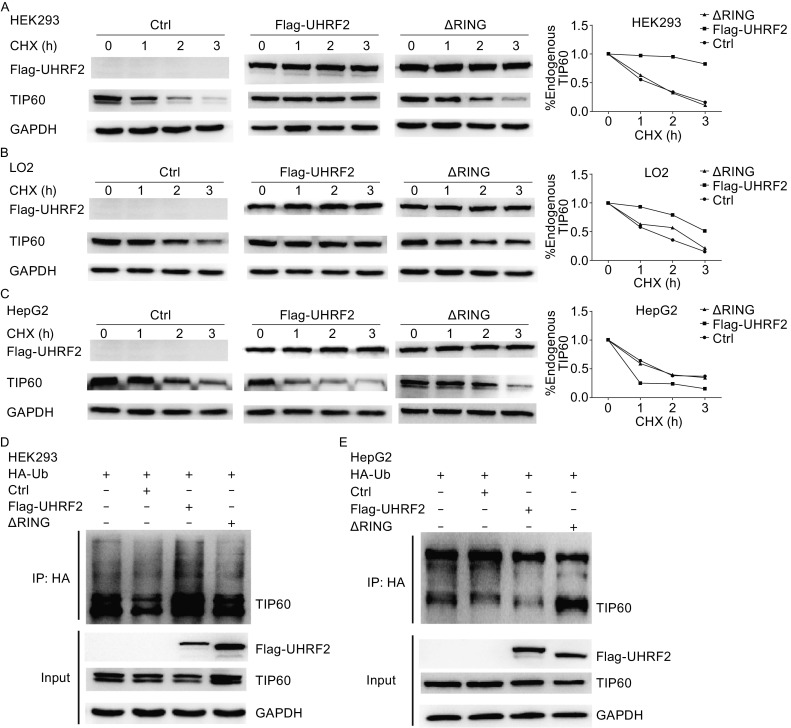
**UHRF2 uniquitinates and stabilizes TIP60** ***in vivo***. (A–C) HEK293, LO2 and HepG2 cells were transfected with control, Flag-UHRF2 or ΔRING plasmids, respectively. The cellular lysates were analyzed by Western blot. Graph represents collated results from three independent half-life experiments performed in HEK293, LO2 and HepG2 cells. TIP60 levels were normalized to 1 at time zero. (D–E) Various plasmids as shown were transfected into HEK293 and HepG2 cells. The cellular lysates were immunoprecipitated with anti-HA antibodies and immunoblotted with anti-TIP60 antibodies

To explore whether UHRF2 stabilizes TIP60, a half-life experiment was performed. Under controlled conditions, TIP60 showed a half-life of less than 1 h, which was extended by UHRF2 in normal cells. Deletion of RING finger domain in UHRF2 abrogated the effect. In cancer cells, the degradation of TIP60 was accelerated by UHRF2 (Fig. 4A–C). These results showed that UHRF2 regulated TIP60 stability via RING finger domain differentially in normal and cancer cells. UHRF2 is an E3 ligase and mediates ubiquitination. In this condition, we hypothesized that UHRF2 might ubiquitinate TIP60 *in vivo*. The results showed that TIP60 was immunoprecipitated with anti-HA-Ub antibodies, and was clearly enhanced in the UHRF2-overexpressing group of HEK293 cells (Fig. 4D). Similar results were not observed in HepG2 cells (Fig. 4E) indicating that UHRF2 stabilized TIP60 expression via RING finger domain through ubiquitination.

### UHRF2 regulates H3K9ac and H3K14ac expression via interaction with TIP60

**Figure 5:**
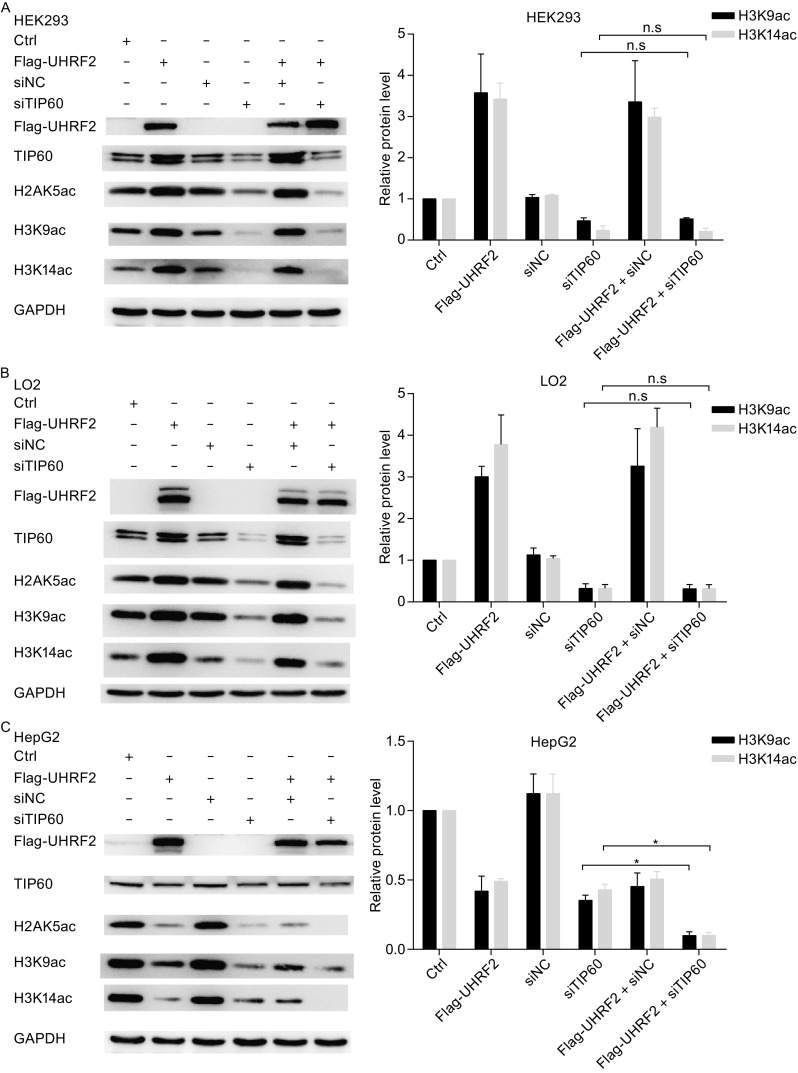

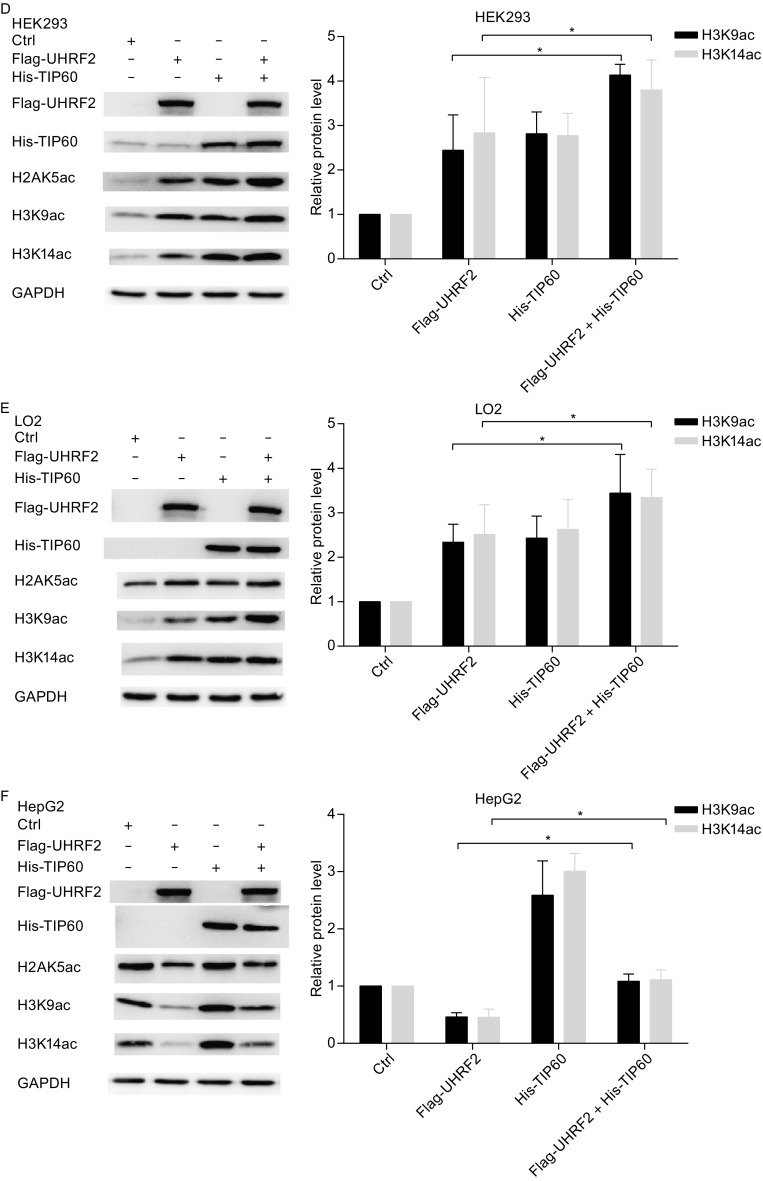

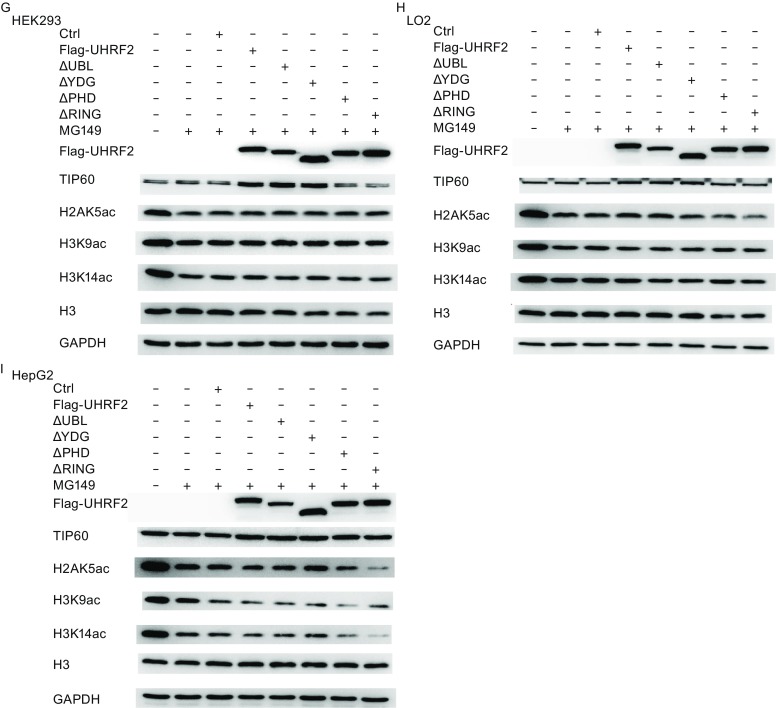
**UHRF2 regulates H3K9ac and H3K14ac via TIP60**. (A–C) HEK293, LO2 and HepG2 cells were transfected with Flag-UHRF2 plasmid, either alone or in combination with siRNA against TIP60. After 48 h, the cellular lysates were analyzed by Western blot. (D–F) HEK293, LO2 and HepG2 cells were transfected with Flag-UHRF2 plasmid, either alone or in combination with His-TIP60 plasmid. Total cellular lysates were analyzed by Western blot. Data represent means ± SD and were examined with two-sided t-test; *P < 0.05; n.s (non-significant) indicated as *P* > 0.05. (G–I) HEK293, LO2 and Hepg2 cells were transfected with various plasmids as shown and treated with MG149 (100 μm) after transfection. Cellular lysates were analyzed by Western blot

Previous studies indicated that TIP60 regulated H3K9ac and H3K14ac expression (Ikura et al., 2015; Renaud et al., 2016; Bassi et al., 2016). In this study, we found similar results in normal and cancer cells (Figure S1). We also suggested that UHRF2 regulated H3K9ac and H3K14ac expression. Hence, we hypothesized that UHRF2 regulates H3K9ac and H3K14ac through TIP60. In Fig. 5A–C, ablation of endogenous TIP60 decreased H3K9ac and H3K14ac protein levels, whereas overexpression of UHRF2 failed to rescue the altered normal cells. In cancer cells, ablation of TIP60 and UHRF2 overexpression further decreased H3K9ac and H3K14ac protein levels. Moreover, co-expression of UHRF2 and TIP60 increased the protein levels of H3K9ac and H3K14ac in normal cells. However, the protein levels of H3K9ac and H3K14ac were not rescued by the co-expression of TIP60 and UHRF2 in cancer cells (Fig. 5D–F). These results clearly showed that TIP60 as a downstream molecule of UHRF2 enhanced the expression of H3K9ac and H3K14ac. We also inhibited TIP60 acetyltransferase activity using a specific inhibitor MG149 that targets acetyl CoA-binding sites. The results showed that inhibition of TIP60 acetyltransferase activity during transfection of full-length UHRF2 or the deletion mutant construct of UHRF2 failed to rescue H3K9ac and H3K14ac expression (Fig. 5G–I). Thus, we concluded that UHRF2 regulated H3K9ac and H3K14ac expression via TIP60 downstream.

### Inverse correlation between TIP60 and UHRF2 in HCC tissues

**Figure 6:**
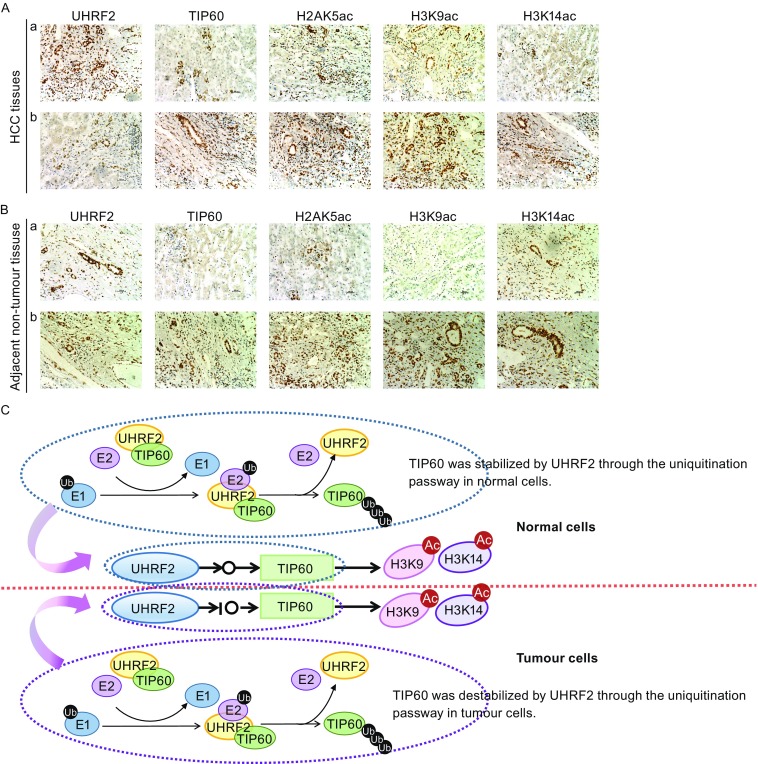
**TIP60 and UHRF2 were negatively correlated in HCC tissues**. (A) Immunohistochemistry with indicated antibodies in HCC tissues. UHRF2 showed a high expression in group “a” and low expression in group “b”. (B) Immunohistochemical analysis using the indicated antibodies in adjacent non-tumor tissues. UHRF2 showed a high expression in group “a” and low expression in group “b”. (C) Model of UHRF2-TIP60-H3K9ac and H3K14ac signaling axis

In this study, we found that UHRF2 regulated H3K9ac and H3K14ac expression via interaction with TIP60 in cultured cells. We investigated the regulatory mechanism in human hepatocellular carcinoma tissues using immunohistochemistry. A significant decrease in the expression of TIP60, H3K9ac, H3K14ac and H2AK5ac was found following the elevated expression of UHRF2 in HCC tissues (Fig. 6A). However, in the adjacent non-tumor tissues, a positive correlation between the aforementioned proteins and UHRF2 was observed (Fig. 6B). Similar results were clearly found in human tissues compared with *in vitro* results. Accordingly, we established that UHRF2 regulated the expression of H3K9ac and H3K14ac by stabilizing TIP60 via proteasomal pathway through its RING domain. We developed a model of UHRF2-TIP60-H3K9ac and H3K14ac signaling axis (Fig. 6C).

## DISCUSSION

TIP60 is a transcriptional co-factor involved in several essential physiological processes in cells. Its aberrant expression has been reported in several cancers (Feng et al., 2016; Mo et al., 2016). TIP60 plays a major role in histone acetylation to potentially trigger cancer-related gene expression (Sun et al., 2015). A previous study showed that TIP60 co-localizes with the UHRF1/DNMT1 complex, which is involved in ubiquitination as a substrate of E3 ligase (Dai et al., 2013). Our findings strongly suggested that TIP60 was a new substrate for UHRF2. Protein modification via ubiquitination is of great importance in many regulatory processes inside the cells (Hershko et al., 2000). Ubiquitination alters protein turnover via proteasome-mediated degradation of proteins (Harrison et al., 2016; Leithe 2016; Yamano et al., 2015; Lechtenberg et al., 2016). Altered ubiquitination may prolong the half-life of specific proteins, while other proteins are rapidly degraded (Vinther-Jensen et al., 2015). In ubiquitination pathway, the E3 ligase plays an important role in substrate recognition. In this study, we found that E3 ligase UHRF2 ubiquitinated TIP60 and further stabilized its expression and elevated its activity via RING finger domain in normal cells. Conversely, the expression and activity of TIP60 were decreased in tumor cells. These results are of utmost significance, with two diametrically opposite responses in different cells. We conjectured that a series of unknown signal transduction factors mediate this pathway, which remain to be investigated.

PTMs associated with gene expression usually occur at the amino acid level, including methylation, phosphorylation, ubiquitination, SUMOylation, plamitoylation, and acetylation (Holt et al., 2015; Kouzarides 2007; Li 2002). Histone acetylation is one of the reversible PTMs catalyzed by HATs and HDACs, which play a key role in the regulation of specific gene expression, chromosome segregation and leukemia (Basu et al., 2009; Wang et al., 2008). Histone acetylation alters the chromosome structure and gene activity, and triggers the initiation and progression of tumors via regulation of cellular proliferation and apoptosis (Pokholok et al., 2005). The proliferation and differentiation levels are balanced under normal conditions through s series of molecular regulatory mechanisms (Tan et al., 2011). Normal cells transform into tumor cells when the balance of histone acetylation and deacetylation is disrupted by physical, chemical and biological factors to trigger abnormal differentiation and chaotic proliferation (Su et al., 2016). Histone H3 is a core component of eukaryotic nucleosome octamer (Karmodiya et al., 2012; Yamada et al., 2013). The two lysine sites 9 and 14 are the focus of intensive study in histone acetylation (Karmodiya et al., 2012). H3K9ac is associated with meiotic recombination hotspots and DNA damage response, and plays a key role in recombination with other factors (Grezy et al., 2016). H3K14ac interacts with transcription factors in tumor initiation and progression (Pichler et al., 2011). In this study, we found that UHRF2 regulated H3K9ac and H3K14ac expression via YDG and RING finger domain, and contrasting results were found in normal and cancer cells. In addition, we found that TIP60 elevated the expression of H3K9ac and H3K14ac. Based on these results, we investigated the UHRF2-mediated regulation of H3K9ac and H3K14ac via interaction with TIP60, and ubiquitination of TIP60 as a new substrate of E3 ligase UHRF2. Interestingly, the differential regulatory mechanism of UHRF2 against TIP60 in normal and cancer cells attracted our attention and investigation.

In summary, we suggest that UHRF2 regulated H3K9ac and H3K14ac expression via the TIP60 downstream signal. It is widely believed that cellular protein-protein interactions are universal. However, in this study, contrasting results were repeatedly found in normal and cancer cells. We used immunohistochemical analysis of HCC tissues and similar results were obtained. The differences in normal and cancer cells were the focus of this study, although we found that an intermediate signaling molecule “TIP60” participated in this mechanism. However, several unknown factors remain to be further investigated. We hypothesized that this mechanism includes specific signal transduction pathways, which trigger carcinogenesis in normal cells, and promote or co-promote malignant transformation of cells.

## MATERIALS AND METHODS

### Cell lines and cell culture

Human normal liver cells (LO2) were cultured in RPMI-1640 medium (Gibco, USA) with 15% fetal bovine serum (Gibco). Human embryonic kidney 293 cells (HEK293) and human hepatocellular liver carcinoma cells (HepG2) were routinely cultured in DMEM/HIGH glucose medium (Hyclone, USA) with 10% fetal bovine serum (Gibco). In this study, HEK293 and LO2 cells were regarded as normal cells. HepG2 cells were regarded as cancer cells.

### Plasmids and siRNAs

Full-length and mutant UHRF2 genes (Flag-UHRF2, ΔUBL, ΔYDG, ΔPHD, ΔRING) were obtained by cloning the PCR fragment corresponding to UHRF2 into the pCMV-3×Flag vector. The pcDNA3.0-HA-Ub was a kind gift obtained from Lu Bai (Fudan University). The pcDNA3.1-His-TIP60, short hairpin RNA (shRNA) against UHRF2 and small interfering RNA (siRNA) against TIP60 were purchased from Invitrogen (USA) and GenePhama (China), respectively. Transfection was performed using Lipofectamine 2000 reagent (Invitrogen) according to the manufacturer’s instructions.

### Western blot

Total protein was isolated with RIPA lysis buffer (Beyotime Biotechnology, China) supplemented with phenylmethanesulfonyl fluoride (PMSF) (Roche, Germany) and a protease inhibitor cocktail (Roche) after 48 h transfection. Protein concentrations were measured using a BCA protein assay Kit (Beyotime Biotechnology) and boiled in loading buffer containing 1% SDS before infection. Proteins were separated by SDS-PAGE followed by Western blot with the indicated antibodies. The primary and secondary antibodies are listed in Table S1.

### Tissue samples and immunohistochemistry

Forty-five cases of HCC tissue samples were collected from patients who underwent surgical resection at the department of the First Affiliated Hospital of Chongqing Medical University. The study was approved by the Ethics Committee of Chongqing Medical University (reference number: 2015018). All subjects signed informed consent. Patients’ information will not be disclosed completely.

Immunohistochemistry was performed as described elsewhere (Das et al., 2016).

### Co-immunoprecipitation

Cells were lysed with NP40 lysis buffer (Beyotime Biotechnology) supplemented with PMSF and a protease inhibitor cocktail at 48 h after transfection. The whole cell extracts were immunoprecipitated overnight at 4°C with the indicated antibody. After extensive washing with lysis buffer, the immune complexes were boiled in a loading buffer containing 1% SDS to deplete the interference of poly-ubiquitin chain on the other molecules in the same complex, and analyzed by Western blot.

### Ubiquitination assay

To detect ubiquitinated TIP60 proteins, cells in a 10 mL cell bottle were transiently transfected with 4 μg HA-ubiquitin-expressing plasmids together with the indicated plasmid. Twelve hours before collection, cells were treated with 20 μmol/L of MG132 (Sigma-Aldrich, USA). Cells were lysed using NP40 lysis buffer supplemented with PMSF and protease inhibitor cocktail. The following procedures were identical to the immunoprecipitation assay except for the use of monoclonal HA antibody.

### Half-life experiments

Cells were treated with 100 μg/mL of cycloheximide (CHX) (ABmole Bioscience, USA) for the indicated times, to inhibit protein synthesis. The cellular lysates were analyzed by Western blot.

### Immunofluorescence

Cells seeded on the coverslips were fixed in 4% paraformaldehyde, and incubated with primary antibodies and secondary antibodies before staining with DAPI, mounted and observed, as described elsewhere (﻿Liang et al., 2016; Takase et al., 2016).

### Statistics analysis

All statistical data are described in the figure legends. Two-sided unpaired Student’s *t*-test was used for experimental comparisons, after quantification with Image J software. Significance was represented as *P < 0.05, and non-significance was indicated as n.s *P* > 0.05.

## Electronic supplementary material

Below is the link to the electronic supplementary material.

Supplementary material 1 (PDF 181 kb)
