# Supplementary material for: E3 ligase UHRF2 stabilizes the acetyltransferase TIP60 and regulates H3K9ac and H3K14ac via RING finger domain
Source: Protein Cell. 2016 Oct 14;8(3):202–18. doi: 10.1007/s13238-016-0324-z (PMC5326618; doi:10.1007/s13238-016-0324-z)
Supplement: Supplementary file 1 — Supplementary material 1 (PDF 181 kb) [file 13238_2016_324_MOESM1_ESM.pdf]

SUPPLEMENTARY MATERIALS

Table S1

Primary and secondary antibodies used for experiments.

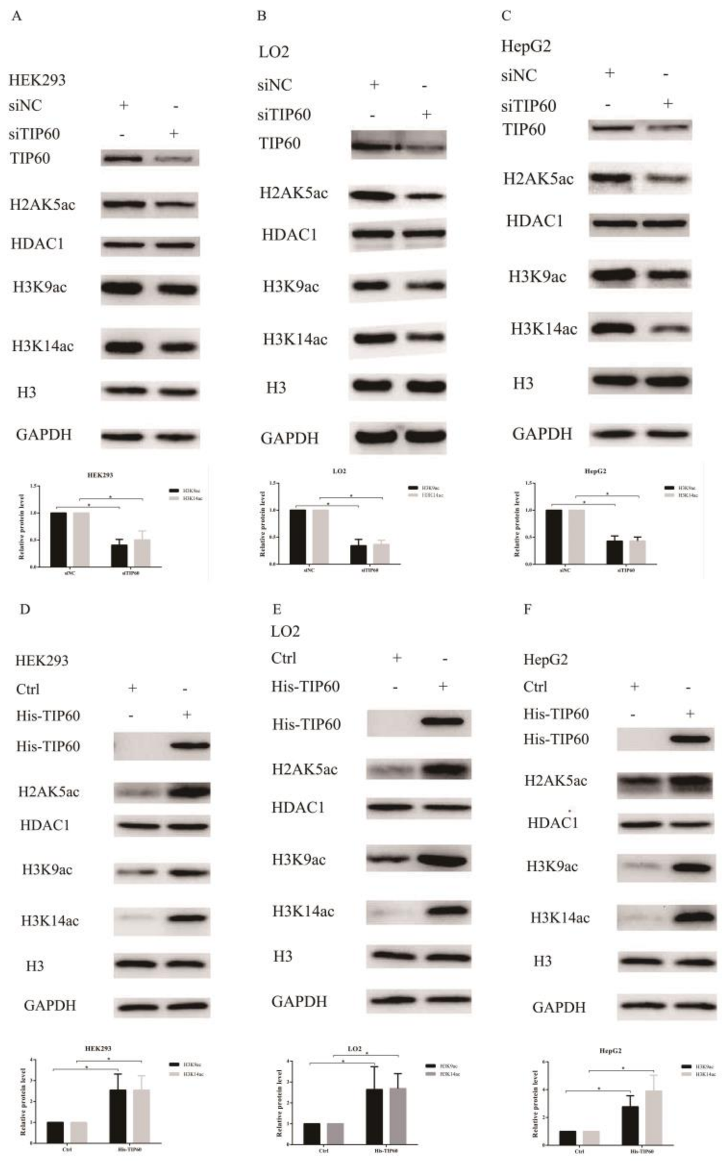

Figure S1

TIP60 regulates the expression of H3K9ac and H3K14ac. (A-C) Endogenous TIP60 was decreased in HEK293, LO2 and HepG2 cells treated with siRNA against TIP60. Total cellular lysates were analyzed by Western blot. (D-F) HEK293, LO2 and HepG2 cells were transfected with His-TIP60 plasmid. After 48 h, the cellular lysates were analyzed by Western blot with the indicated antibodies. The signals were quantified using Image J software and the data were analyzed with two-sided t-test. *$p < 0.05$; and n.s (nonsignificant) indicates $p > 0.05$.
